# Evaluation of Antioxidant Potentials and *α*-Amylase Inhibition of Different Fractions of Labiatae Plants Extracts: As a Model of Antidiabetic Compounds Properties

**DOI:** 10.1155/2017/7319504

**Published:** 2017-09-10

**Authors:** Soheila Moein, Elham Pimoradloo, Mahmoodreza Moein, Mahmood Vessal

**Affiliations:** ^1^Molecular Medicine Research Center, Hormozgan Health Institute, Hormozgan University of Medical Sciences, Bandar Abbas, Iran; ^2^Department of Biochemistry, Faculty of Medicine, Hormozgan University of Medical Sciences, Bandar Abbas, Iran; ^3^Department of Biochemistry, Fars Sciences and Research Branch, Islamic Azad University, Shiraz, Iran; ^4^Medicinal Plants Processing Research Center, Shiraz University of Medical Sciences, Shiraz, Iran; ^5^Department of Pharmacognosy, School of Pharmacy, Shiraz University of Medical Sciences, Shiraz, Iran; ^6^Department of Biochemistry, Faculty of Basic Sciences, Islamic Azad University of Shiraz, Sadra City, Fars, Iran

## Abstract

In an attempt to identify herbal drugs which may become useful in the prevention of diabetes, antioxidant potentials and *α*-amylase inhibition by the ethanol extracts of two plants belonging to Lamiaceae family,* Otostegia persica* and* Zataria multiflora*, and their different fractions were studied. Also, inhibition of *α*-amylase by* Salvia mirzayanii* and its fractions was evaluated. All of the samples exhibited antioxidant activities, among which ethyl acetate fraction of* Zataria multiflora* (17.21 ± 0.17 mg GAE/g) was found to contain the highest amounts of phenols and the ethyl acetate fraction of* Zataria multiflora* (218 ± 2.76 mg QUE/g) had the most values of flavonoids. Ethyl acetate fraction of* Zataria multiflora* (IC_50_ = 3.05 ± 0.51 *μ*g/ml) was shown to have the most reducing power and the ethyl acetate fraction of* Zataria multiflora* (IC_50_ = 32.17 ± 1.82 *μ*g/ml) exhibited the highest DPPH radical scavenging. The ethyl acetate fraction of* Otostegia persica* (99.39 ± 0.94%) showed the highest *α*-amylase inhibitory activity which was similar to acarbose used as a standard. Mode of *α*-amylase inhibition of the most samples was uncompetitive except for* ZMC*,* OPP*,* OPC*, and* SMP* which presented competitive inhibition. The present findings showed that studied samples may have some compounds with antioxidant and antidiabetic effects.

## 1. Introduction

Type 2 diabetes (T2D) affects a large population worldwide. T2D is a complex heterogeneous group of metabolic disorders including hyperglycemia and impaired insulin action and/or insulin secretion and causes dysfunctioning of multiple organs or tissues. Current theories of T2D include a defect in insulin-mediated glucose uptake in muscle, a dysfunction of the pancreatic *β*-cells, a disruption of the secretory function of adipocytes, and an impaired insulin action in the liver [1]. The early stage of type 2 diabetes is associated with postprandial hyperglycemia due to impaired pancreas after meal.

Hyperglycemia is believed to increase the production of free radicals and reactive oxygen species, leading to oxidative tissue damage and diabetic complications such as nephropathy, neuropathy, retinopathy, and memory impairment [2].

Glucosidases are a group of digestive enzymes which break down the dietary carbohydrates into simple monosaccharides. Glucosidase inhibitors such as acarbose reduce the rate of carbohydrate digestion and delay the carbohydrate absorption from the digestive tract; therefore, they have a potential to prevent the development of type 2 diabetes by lowering the after meal glucose levels [3]. Natural compounds from ancient times have been regarded as the most important sources of supplying drugs and now half of the drugs are from natural origin. In recent years, because of the availability of these compounds, having renewable resources, low toxicity, and low cost for preparing them [4], there is a tendency to isolate active compounds from natural sources. Labiatae family (Lamiaceae) is one of the largest and most distinctive families of flowering plants, with about 220 genera and almost 4000 species worldwide. This family has an almost cosmopolitan distribution; moreover, Labiatae family is best known for the essential oils common to many members of the family. Many biological active essential oils have been isolated from various members of this family; in addition to, Labiatae family is also famous for the presence of diterpenoids [5] and polyphenolic compounds [6]. “Antioxidant compounds such as polyphenols are common in plants; however, the most concentrations of these components are present specially in fruits such as apples, grapes, blackberries, and strawberries [7]. These components can scavenge free radicals and exert protective effects against cardiovascular diseases [7].”

In previous studies, polyphenolic compounds were reported to be strong *α*-amylase inhibitors [8]. To achieve this, antidiabetic (inhibition of *α*-amylase) and antioxidant properties of two plants extracts and their fractions were investigated. These two plants include* Otostegia persica (Op)* and* Zataria multiflora (Zm)* which belong to Labiatae family. Also, inhibition of *α*-amylase by* Salvia mirzayanii (Sm)* extract and its different fractions will be investigated. This plant belongs to Labiatae family.

## 2. Materials and Methods

### 2.1. Chemicals

DPPH (2,2-diphenyl-1-picrylhydrazyl radical), quercetin, gallic acid, *α*-amylase and Folin-Ciocalteu reagent were obtained from Sigma Chemical Co. (St Louis, MO). All other reagents were obtained from Merck Chemicals (Darmstadt, Germany).

### 2.2. Plant Materials

The leaves of* Op* and* Sm* were collected from Genu Mountains in the northeast of Bandar Abbas on 20 and 15 June 2012, while* Zataria multiflora* was obtained from a herbalist in Shiraz, June 2012; after that, the plants were identified by Dr. M. Moein. The voucher deposited in Medicinal Plants Processing Research Center, School of Pharmacy, Shiraz University of Medical Sciences. The voucher numbers of* Op, Zm*, and* Sm* are MPRCM 94-85, MPRCM 94-83, and MPRCM 94-84, respectively.

### 2.3. Extraction and Fractionation

The leaves were separated, dried at room temperature, and were ground into a powder form; then, using a mixture of 70% ethanol in water, hydroalcoholic extracts of the powdered leaves were prepared by a percolation method and the extracts were subjected to fractionation by different solvents in order of increasing polarity (petroleum ether, chloroform, ethyl acetate, and butanol), respectively. The extracts and their fractions were concentrated by a rotary vacuum evaporator and kept at +4°C until tested.

### 2.4. Determination of Total Phenols

Total phenol compound contents were determined by Folin-Ciocalteu method [9]. To start the reaction, 150 *μ*l of samples (10 mg/ml) was mixed with 750 *μ*l of Folin-Ciocalteu reagent 0.2 N and, after 3 min, 600 *μ*l of Na_2_CO_3_ (7.5%) was added. The mixture was put at room temperature in the dark for 30 min. The phenolic contents were determined by colorimetric method at 765 nm. Gallic acid was used as a standard and the total phenolic content in gallic acid equivalent (GAE) was calculated by the following formula:

(1) $$\begin{array}{c} C=\frac{cv}{m}, \end{array}$$

where *C* is the total content of phenolic compounds (mg/g plant extract or its fraction) in GAE; *c* is the concentration of gallic acid established from the calibration curve (mg/ml); *v* is the volume of extract (ml); and *m* is the weight of plant extract or its fractions.

### 2.5. Determination of Flavonoids Content

The flavonoid contents of* Zm* and* Op* extracts and their fractions were determined, based on a colorimetric assay [10] in which 0.5 ml of different concentrations of quercetin (12.5–300 *μ*g/ml) was used as a standard. Quercetin solutions were mixed with 2 ml of deionized water, and, after 3 min, 150 *μ*l of NaNO_2_ (5% w/v) and 150 *μ*l AlCl_3_ (10% w/v) solutions were added, and, after 6 min, 2 ml of NaOH (4% w/v) was also added to the mixture and the volume of the assay mixture was raised to 5 ml with distilled water. After vortexing, the mixture was incubated at room temperature for 15 min and its absorbance was measured at 510 nm. Flavonoid content was determined as mg of quercetin equivalent, using an equation obtained from the standard quercetin calibration curve.

### 2.6. Determination of Reducing Power

The reducing power of the samples was determined according to the method of Duan et al. [11]. Briefly, 0.5 ml of different concentrations of the samples (2.5 to 40 *μ*g/ml in ethanol) was mixed with 1.25 ml of 0.1 M phosphate buffer (pH 6.6) and 1.25 ml of a 1% (w/v) solution of potassium ferricyanide; then, the mixture was incubated in a water bath at 50°C for 20 min. Afterwards, 1.25 ml of 10% (w/v) trichloroacetic acid solution and 1.25 ml of distilled water and 0.25 ml of 0.1% (w/v) solution of ferric chloride were added to 2.5 ml of the mixture. Immediately the absorbance of the samples was measured at 700 nm. Increase in the absorbance of the reaction mixture indicated increase in the reducing power. Gallic acid was used as a standard. The experiments were repeated three times and their averages were reported. The least concentration of the sample that exhibited OD = 0.5 was reported.

### 2.7. Determination of DPPH Radical Scavenging

The DPPH scavenging assay was performed according to procedures described by Moein et al. [12].

One-tenth ml of the samples with different concentrations (12.5–800 *μ*g/ml) was mixed with 0.1 ml of DPPH solution (0.5 mM in methanol), incubated at 25°C in the dark for 30 min, and the absorbance was measured at 490 nm using a microplate reader (Biotek, ELX 800). Quercetin was used as an antioxidant standard. The obtained data was used to determine the concentration of the sample required to scavenge 50% of the DPPH free radicals (IC_50_). The percent inhibition was plotted against the concentrations of the extract and IC_50_ was obtained from the fitted linear curve. A lower IC_50_ demonstrates a more potent antioxidant potential. The results were expressed as the mean ± SD of three replicates.

### 2.8. Determination of *α*-Amylase Inhibition

*α*-Amylase inhibitory activities of* Op*,* Sm*, and* Zm* extracts and their fractions were performed according to Ademiluyi et al. [13] with some modifications. Briefly starch solution (2 mg/ml) was obtained by boiling and stirring 20 mg of starch in 10 ml of 10 mM sodium phosphate buffer (pH 6.9) for 15 min. The enzyme solution (4 unit/ml) was prepared by mixing 0.001 g of *α*-amylase (EC 3.2.1.1) in 1.5 ml of the mentioned buffer. The extracts (3.7 mg) were dissolved in 0.5 ml ethanol to give concentration about 7.4 mg/ml. Dinitrosalicylic acid (DNSA) was used as a color reagent. Stop solution contained 96 mM 3,5-dinitrosalicylic acid (10 ml) and 5.31 M sodium potassium tartrate in 2 M sodium hydroxide (10 ml). To the plant extract (10 *μ*l), 550 *μ*l of starch solution and 40 *μ*l of the enzyme solution were added in a tube, mixed and incubated at 37°C for 15 min; then, 600 *μ*l of DNSA was added to this mixture and the tube was incubated at 85°C in a water bath. After 15 min, the reaction mixture was removed from water bath and cooled. The absorbance was measured at 540 nm in a spectrophotometer (Kyoto, Japan). Individual blanks were prepared for correcting the background absorbance. Controls were conducted in an identical fashion, by replacing plant extracts with 1 ml of ethanol. Acarbose was used as the positive control. *α*-Amylase inhibitory activity was calculated as follows:

(2) $$\begin{array}{c} \text{Inhibition }\left(\;\%\;\right)=100-\left(\;\frac{{OD}_{\text{samp-blank}}}{{OD}_{\text{cont-blank}}}\times 100\;\right), \end{array}$$

where OD samp and OD cont were defined as the absorbance of the sample and the control, respectively.

### 2.9. Determination of Kinetic Parameters of *α*-Amylase Inhibition

Inhibition patterns of the samples against *α*-amylase were determined according to the method described by Kim et al. [14]. Briefly, a fixed amount of *α*-amylase was incubated with increasing concentrations of starch as a substrate (1–6 mg/ml) at 37°C for 15 min, in the absence or presence of the samples (7.4 mg/ml).

Reactions were terminated and the absorption measurements carried out as mentioned before. Amounts of the product liberated (maltose) were determined from corresponding standard curves and converted to reaction rates. Inhibition patterns were then determined by Lineweaver-Burk plot (1/*v* versus 1/(*S*)).

### 2.10. Statistical Analysis

All experiments were performed in triplicate and the data were expressed as mean ± SD (standard deviation). Linear regression was performed for calculation 50% inhibitory concentration (IC_50_). Microsoft EXCEL 2010 program and graph pad instate 3.0 software were used for data analysis. One-way analysis of variance (ANOVA) was used to assess the presence of significant differences (*p* < 0.01).

## 3. Results

### 3.1. Quantification of Phenolic Compounds

Total phenols and flavonoids contents of the plant extracts are presented in Table 1. The results of phenol assay showed that, among* Zm* extract and its fractions,* ZMA* and* ZMP* had the most and the least amounts of phenolic compounds, respectively. Among* Op* extract and its fractions,* OPA* and* OPP* had the most and the least amounts of phenolic compounds. In general, the results of statistical analysis showed that there are significant differences between phenolic compounds in all of the samples (*p* ≤ 0.05).

The results about the flavonoids showed that, among* Zm* extract and its fractions, the highest amounts of flavonoids and the least amounts of flavonoids were in* ZMA* and* ZMP*, respectively. Among* Op* extract and its fractions, the highest and the least amounts of flavonoids were found in* OPP* and* OPC*, respectively, as shown in Table 1.

### 3.2. Determination of Antioxidant Activities

Table 2 reveals the reducing power and radical (DPPH) scavenging power of* Zm* and* Op* extracts and their fractions. The results of DPPH radical scavenging were reported as IC_50_, which show the concentrations of the samples which scavenge DPPH radical 50%, and, in reducing power, the concentrations of the samples having an OD = 0.5 at 700 nm were reported.

**Figure 1:**
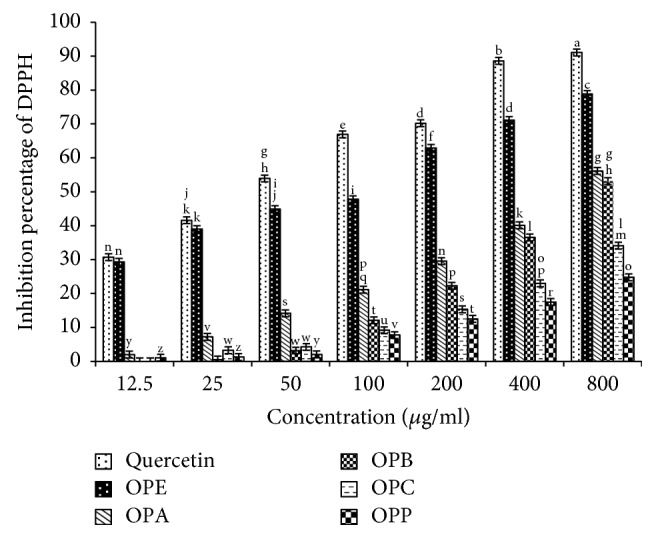
DPPH radical scavenging of* Otostegia persica* extract and its fractions in comparison with quercetin as an antioxidant standard.

**Figure 2:**
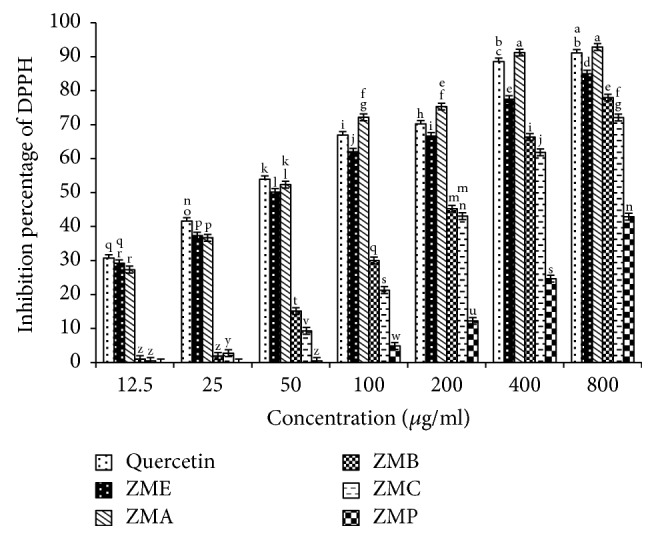
DPPH radical scavenging of* Zataria multiflora* extract and its fractions in comparison with quercetin as an antioxidant standard.

Figures 1 and 2 show the DPPH radical scavenging of various concentrations of* Zm* and* Op* extracts and their fractions in comparison with quercetin as a standard.

**Figure 3:**
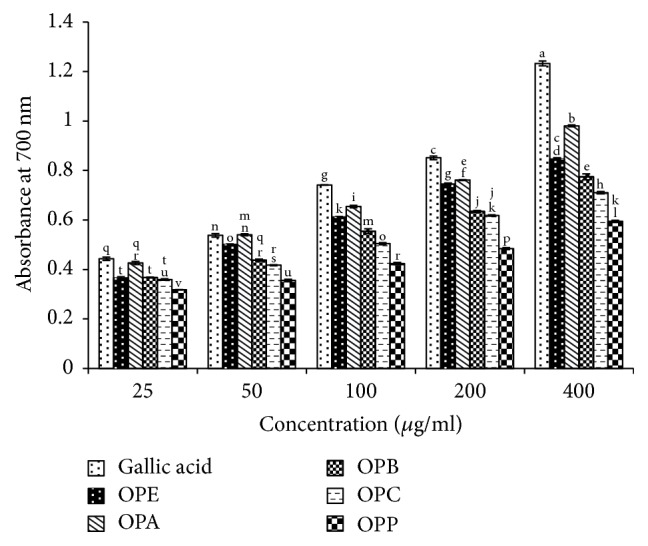
Reducing power of* Otostegia persica* extract and its fractions in comparison with gallic acid as an antioxidant standard.

**Figure 4:**
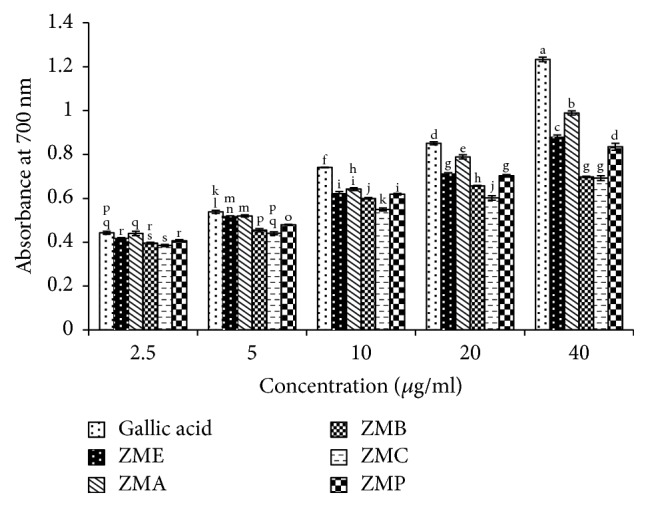
Reducing power of* Zataria multiflora* extract and its fractions in comparison with gallic acid as an antioxidant standard.

Figures 3 and 4 present reducing power of various concentrations of* Zm* and* Op* extracts and their fractions in comparison with gallic acid as a standard.

### 3.3. Determination of *α*-Amylase Inhibition

Alcoholic extracts of* Zm* and* Op (ZME* and* OPE*, resp.) and their different fractions were tested for *α*-amylase inhibition using a colorimetric method as mentioned in methods. *α*-Amylase inhibitory potentials of the extracts and their fractions were compared with acarbose as a standard under our specific set of assay conditions. As shown in Table 3, all of the samples except* ZMP* had inhibitory effects on *α*-amylase.

### 3.4. Type 2 Diabetes of *α*-Amylase Inhibition by the Samples

**Figure 5:**
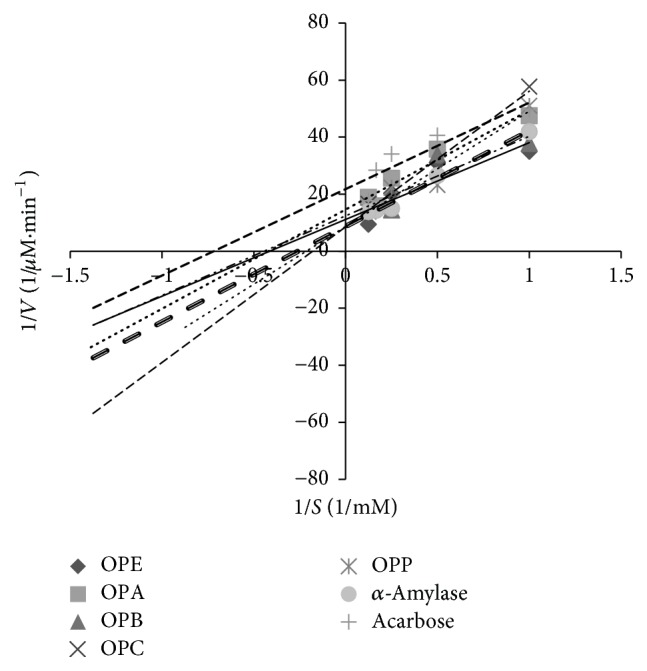
Kinetic analysis of *α*-amylase inhibition by* Otostegia persica* extract and its fractions.

**Figure 6:**
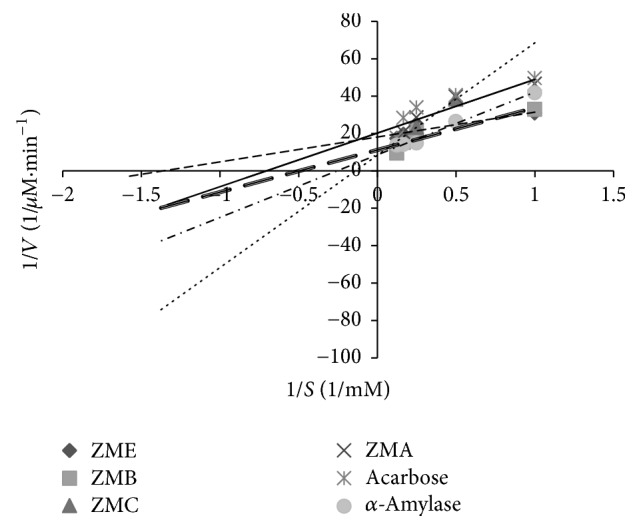
Kinetic analysis of *α*-amylase inhibition by* Zataria multiflora* extract and its fractions.

**Figure 7:**
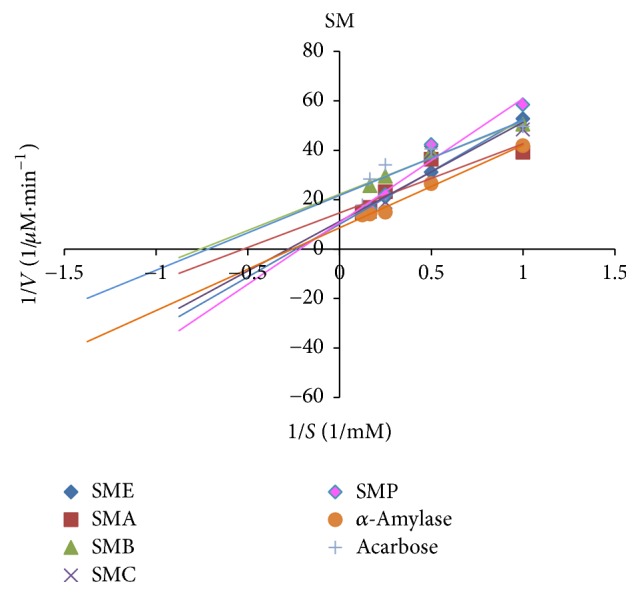
Kinetic analysis of *α*-amylase inhibition by* Salvia mirzayanii* extract and its fractions.

Table 3 demonstrates the *K*_*m*_ and *V*_max_ values of the samples against *α*-amylase. As compared to the uninhibited reaction, a decrease in *V*_max_ value was noted for the reactions with* ZME*,* ZMB*,* ZMC*,* OPE*,* OPA*, and* OPB* and no change in *V*_max_ value was noted in the reactions for* ZMC*,* OPC*, and* OPP*. The effects of the samples on *K*_*m*_ were different too. This means that *K*_*m*_ was constant in the reaction with* ZMC*,* OPC*, and* OPP* and decreases in other samples. The types of inhibition of porcine pancreatic *α*-amylase by different extracts and fractions and also by acarbose as a positive standard were obtained from Lineweaver-Burk plot (Figures 5, 6, and 7). The results suggested that the type of inhibition by acarbose and all of the fractions except* ZMC*,* OPC*, and* OPP* was uncompetitive. In* ZMC*,* OPC*, and* OPP* inhibition of *α*-amylase may be competitive.

## 4. Discussion

“In an effort for identifying herbal drugs which may become useful in the prevention or alleviation of diabetes [15],” this study is performed. Herbal plants have long been used to treat diabetes, as their principal bioactive components showed good antidiabetic and antioxidant properties [16]. Many herbal plant extracts have been reported for their *α*-amylase inhibitory activities but to date no such report was found for* Op, Sm*, and* Zm* extracts. These *α*-amylase inhibitors are also called starch blockers since they prevent or slow down the absorption of starch into the body mainly by blocking the hydrolysis of 1,4-glycosidic linkages of starch and other oligosaccharides into maltose, maltotriose, and other simple sugars [17]. Based on our research, our study is the first report to state *α*-amylase inhibition by leaves extracts of* Op*,* Sm*, and* Zm* and their fractions. It is shown that potency of *α*-amylase inhibition is related to the presence of certain compounds such as tannins, phenols, flavonoids, and compounds with antioxidant activities [18, 19]. So we investigated the presence of the phenolic compounds and flavonoids and the antioxidant powers of the samples with two methods including DPPH radical scavenging and reducing power. The antioxidant potentials of* Sm* extract and its fractions were reported in other study [20].

In our study,* OPA* showed the highest inhibition of *α*-amylase (99.4 ± 0.94%, Table 3) activity which could be attributed to the presence of polyphenols (7.072 ± 0.11 mg GAE/g sample, Table 1) and flavonoids (40.37 ± 1.06 mg QUE/g sample, Table 1). Not only are polyphenols capable of reducing oxidative stress but also they inhibit carbohydrate hydrolyzing enzymes because of their abilities for binding proteins [21, 22]. Our results are in accordance with a previous study, in which there was a positive correlation between total polyphenols and flavonoids contents and the ability to inhibit intestinal *α*-glucosidases and pancreatic *α*-amylase [21–24]. In our study, acarbose was used as the positive control; it inhibits the *α*-amylase activity at a concentration of 7.4 mg/ml, 100%, and using the same concentration of* OPA*, an inhibition of about 99% was obtained. This indicates that* OPA* is very potent in *α*-amylase inhibition. This could justify the nature of some extract constituents (phenols, flavonoids saponins, steroids, alkaloids, and terpenoids) which are present in the extract and effective inhibitors of *α*-amylase [25, 26]. Oxidative stress plays a pivotal role in the development of diabetic complications. Free radicals are formed disproportionately during diabetes due to glucose oxidation and the subsequent oxidative degradation of glycated proteins [2]. In addition, the diabetic patients have enhanced cellular oxidative stress and reduced antioxidant potentials which lead to defective antioxidant status [27]. In the present study, also the antioxidant capacities (by DPPH radical scavenging in Figures 1 and 2 and reducing power in Figures 3 and 2) and the presence of flavonoids and polyphenolics have been evaluated that could contribute to hypoglycemic activities. The effect of antioxidants on DPPH radical scavenging was thought to be due to their hydrogen-donating ability [9]. The correlation between antioxidant activity and the content of phenolic compounds has also been reported by many researchers. We find that the highest phenolic compounds and flavonoids exist in* ZMA* (17.21 ± 0.17 mg GAE/g sample, 218.19 ± 2.76 mg QUE/g sample, resp.) and it has the highest reducing power and radical scavenging with 55.5 ± 1.52%  *α*-amylase inhibitory power. The least amounts of phenolic compounds and flavonoids were found in* ZMP* (2.14 ± 0.03 mg GAE/g sample and 5.83 ± 0.20 mg QUE/g sample, resp.). Also, the lowest reducing power with no antidiabetic *α*-amylase inhibition is observed in this fraction of* Zm*. Among extract and different fractions of* OP*,* OPA* had the highest phenolic compounds (7.07 ± 0.11 mg GAE/g sample) and flavonoids (40.37 ± 1.06 mg QUE/g sample) and the highest reducing power with (99.4 ± 0.94%) *α*-amylase inhibitory power (Table 3). The lowest phenolic compounds and flavonoids were in found in* OPC* (0.90 ± 0.032 mg GAE/g sample and 24.25 ± 1.24 mg QUE/g sample, resp.). Ethanol extract of* OP* had the highest flavonoids (57.07 ± 1.03 mg QUE/g sample) and other workers have also found that phenolic compounds such as flavonoids which have the ability to scavenge free radicals are in the polar fractions of plant extracts [28]. Generally,* Zm* fractions possess notably high phenolic and flavonoid compounds in comparison with* OP* fractions (*p* ≤ 0.05). Abdali et al. described that the presence of thymol and carvacrol in* Zm* is responsible for its antioxidant activity [29]. There are many reports on the antioxidant and antidiabetic effects of* Zm* and* Op* which agree with our results [16, 26, 30–33]. Lineweaver-Burk plot is frequently used for identification of the likely mechanism of enzyme inhibition [34]. Plots were prepared by the reciprocal of product formation rate (1/*V*0) versus the reciprocal of substrate concentrations. The initial velocities (*V*0) were determined from the slope of the linear parts of the curves of product amounts, in terms of mM of maltose equivalents, versus time in minutes (data not shown). Here, Lineweaver-Burk plot was depicted for both samples and acarbose as the standard inhibitor of porcine pancreatic *α*-amylase (PPA). Acarbose is a strong competitive inhibitor of *α*-amylase. This compound has a pseudosugar ring and the glycosidic nitrogen linkage that mimics the transition state for the enzymatic cleavage of glycosidic bond and hence competitively inhibits *α*-amylase [34]. In our study, Lineweaver-Burk plots of the extracts showed different patterns. For the first time (Figures 5–7), the mechanisms of *α*-amylase inhibition of* Zm*,* Sm*, and* Op* extracts and their different fractions were investigated and the results showed that some of the fractions inhibited *α*-amylase through uncompetitive mechanism (like* ZMA, ZME*,* ZMB*,* OPE*,* OPA*, and* OPB*) and* ZMC, OPP*,* OPC*, and* SMP* inhibited *α*-amylase through competitive mechanism. Probably, there are some compounds in the samples that could compete with the substrate for binding to the active site of the enzyme and some of them do not compete with the substrate but bind to the other sites of the enzyme. In this study, most of the samples are uncompetitive inhibitors and one advantage of these inhibitors is that they would be effective at lower concentrations of substrate in comparison with competitive inhibitors which require higher concentrations of the substrate for the same effects.

## 5. Conclusion

The findings of this study clarify that phenolic compounds present in leaves extracts of* Zm* and* Op* species may be responsible for their observed activities. Thus a few traditional Iranian medicinal plants, particularly* Zm*,* Sm*, and* Op*, seem to act as potential *α*-amylase inhibitors in the management of diabetes.

## Figures and Tables

**Table 1:** Total phenolic compounds and flavonoid contents of the plant extracts and their fractions (*p* ≤ 0.05).

Samples	Total phenol content (mg GAE/g sample)	Total flavonoids content (mg QUE/g sample)
Ethanol extractof *Zm*	3.01 ± 0.07^d^	64.73 ± 1.66^d^
Petroleum ether fraction of* Zm*	2.14 ± 0.03^e^	49.14 ± 0.91^e^
Chloroform fraction of *Zm*	8.01 ± 0.06^c^	81.06 ± 1.89^c^
Ethyl acetate fraction of *Zm*	17.21 ± 0.17^a^	218.19 ± 2.76^a^
Butanol fraction of *Zm*	12.28 ± 0.12^b^	112.88 ± 2.76^b^

Ethanol extract of *Op*	5.21 ± 0.118^b^	57.07 ± 1.03^b^
Petroleum ether fraction of* Op*	0.68 ± 0.02^e^	73.51 ± 0.5^a^
Chloroform fraction of *Op*	0.90 ± 0.032^d^	24.25 ± 1.24^e^
Ethyl acetate fraction of *Op*	7.07 ± 0.11^a^	40.37 ± 1.06^c^
Butanol fraction of *Op*	2.81 ± 0.033^c^	38.29 ± 1.07^d^

*Zm: Zataria multiflora, Op: Otostegia persica*; statistical differences shown as alphabetic letters. The same alphabetic letters implied there are no statistical differences, *p* > 0.05, and different letters represented statistical differences, *p* < 0.05.

**Table 2:** Antioxidant activities of *Zataria multiflora* and *Otostegia persica* extracts and their fractions.

Samples	DPPH radical scavenging (IC_50_ *μ*g/ml)	Reducing power (concentrations with absorbance 0.5)
Ethanol extractof *Zm*	90.73 ± 2.92^c^	4.15 ± 0.013^c^
Petroleum ether fraction of* Zm*	883.26 ± 2.18^f^	5.83 ± 0.20^d^
Chloroform fraction of *Zm*	440.75 ± 3.09^e^	11.36 ± 0.07^f^
Ethyl acetate fraction of *Zm*	32.17 ± 1.82^b^	3.05 ± 0.51^b^
Butanol fraction of *Zm*	393.78 ± 1.55^d^	7.57 ± 0.24^e^

Ethanol extract of *Op*	166.09 ± 2.11^b^	64.33 ± 1.15^c^
Petroleum ether fraction of* Op*	1578.89 ± 4.23^f^	248.57 ± 2.85^f^
Chloroform fraction of *Op*	1139.5 ± 3.11^e^	130.74 ± 2.31^e^
Ethyl acetate fraction of *Op*	635.65 ± 3.67^c^	38.66 ± 2.08^b^
Butanol fraction of *Op*	691.32 ± 4.71^d^	103.66 ± 1.52^d^
Quercetin	26.28 ± 2.68^a^	ND
Gallic acid	ND	2.75 ± 0.261^a^

ND: nondetermined, Zm: *Zataria multiflora*, Op: *Otostegia persica*; statistical differences shown as alphabetic letters. The same alphabetic letters implied there are no statistical differences, *p* > 0.05, and different letters represented statistical differences, *p* < 0.05.

**Table 3:** Percentage inhibition and kinetic parameters of *α*-amylase in plant extracts and their fractions.

Samples	*Km* mM	*V*max/min	*α*-Amylase inhibition%
*α*-Amylase without inhibitor	4.2 ± 0.004	0.12 ± 0.02	
Acarbose	0.95 ± 0.02	0.04 ± 0.001	100

Ethanol extract of *Zm*	0.66 ± 0.01	0.05 ± 0.01	47.23 ± 1.2
Petroleum ether fraction of *Zm*	ND	ND	ND
Chloroform fraction of *Zm*	7.2 ± 0.02	0.01 ± 0.001	42.7 ± 0.69
Ethyl acetate fraction of *Zm*	1.4 ± 0.03	0.05 ± 0.001	55.5 ± 1.52
Butanol fraction of *Zm*	0.66 ± 0.01	0.05 ± 0.001	47.23 ± 1.2

Ethanol extract of *Op*	2.42 ± 0.01	0.09 ± 0.001	55.6 ± 0.25
Petroleum ether fraction of *Op*	4.85 ± 0.02	0.12 ± 0.005	53.3 ± 2.5
Chloroform fraction of *Op*	5.7 ± 0.0	0.12 ± 0.0005	58.5 ± 1.4
Ethyl acetate fraction of *Op*	2.4 ± 0.03	0.07 ± 0.001	99.4 ± 0.94
Butanol fraction of *Op*	2.23 ± 0.3	0.08 ± 0.005	65.3 ± 0.7

Ethanol extract of *Sm*	1.96 ± 0.01	0.07 ± 0.004	79.4 ± 1. 3
Petroleum ether fraction of *Sm*	4.6 ± 0.004	0.092 ± 0.01	42.08 ± 0.84
Chloroform fraction of *Sm*	3.6 ± 0.005	0.09 ± 0.03	24.2 ± 1.2
Ethyl acetate fraction of *Sm*	0.43 ± 0.03	0.01 ± 0.03	38.14 ± 1.04
Butanol fraction of *Sm*	1.2 ± 0.02	0.04 ± 0.02	45.6 ± 0.9

*Zm*: *Zataria multiflora*, *Op*: *Otostegia Persica*, *Sm*: *Salvia mirzayanii*, NA: nonactive.
